# Comparisons of tri-ponderal mass index and body mass index in discriminating hypertension at three separate visits in adolescents: A retrospective cohort study

**DOI:** 10.3389/fnut.2022.1028861

**Published:** 2022-10-17

**Authors:** Jia Hu, Yi Zhong, WenXin Ge, Huiling Lv, Ziyao Ding, Di Han, Bo Hai, Hui Shen, Jieyun Yin, Aihua Gu, Haibing Yang

**Affiliations:** ^1^State Key Laboratory of Reproductive Medicine, School of Public Health, Nanjing Medical University, Nanjing, China; ^2^Suzhou Institute of Advanced Study in Public Health, Gusu School, Nanjing Medical University, Suzhou, China; ^3^Suzhou Center for Disease Prevention and Control, Suzhou, China; ^4^Jiangsu Key Laboratory of Preventive and Translational Medicine for Geriatric Diseases, School of Public Health, Medical College of Soochow University, Suzhou, China; ^5^Institute of Child and Adolescent Health, School of Public Health, Peking University, Beijing, China

**Keywords:** adolescents, body mass index, hypertension, pediatric, tri-ponderal mass index

## Abstract

**Objective:**

To estimate whether the new obesity indicator tri-ponderal mass index (TMI) has a better capacity to predict adolescent hypertension (HTN) and HTN subtypes at three separate blood pressure (BP) visits than the conventionally used body mass index (BMI).

**Methods:**

A total of 36,950 adolescents who had initial normal BP from 2012 to 2019 were included in Suzhou, China. HTN was defined as having three separate visits of elevated BP in 2020. The area under the receiver-operating characteristic curve (AUC), false-positive rate, false-negative rate, total misclassification rates, net reclassification improvement (NRI), and integrated discrimination improvement were calculated to compare the discriminative ability of HTN between BMI and TMI.

**Results:**

TMI had better predictive abilities than BMI among all of the participants when predicting HTN (difference in AUC = 0.019, 95% CI = 0.007–0.031; NRI = 0.067, 95% CI = 0.008–0.127) and isolated systolic hypertension (difference in AUC = 0.021, 95% CI = 0.005–0.036; NRI = 0.106, 95% CI = 0.029–0.183). The difference in prediction abilities between BMI and TMI was more obvious in the subgroup of age ≥16. Also, TMI outperformed BMI in predicting adolescent HTN in girls but not in boys.

**Conclusion:**

Compared with BMI, TMI may have a better predictive capacity for HTN, particularly in girls and older adolescents. TMI has the potential to be used as an effective predictor for HTN in clinic practice. Further studies are needed to verify the utility of TMI.

## Introduction

Hypertension (HTN) is a widespread chronic disease that receives increased global health attention, especially in pediatric populations (1, 2). The prevalence of HTN among children aged 6–19 has increased by 75–79% in the past 15 years (2). Nevertheless, the diagnosis rate of adolescent HTN needs to be improved. In Europe and the US, only 13–26% of children with HTN were properly identified (3). Additionally, adolescent HTN has been identified as an established risk factor for cardiovascular disease (4–6), organ damage (7), and premature death (8), and may turn into adulthood HTN (9–11). Therefore, to reduce the potential burden of disability and premature death, early and precise detection of HTN in children and adolescents is in need.

The measurement of HTN in children and adolescents is more unstable and complicated than in adults (2) due to regression to the mean (12), the “white-coat” effect (13), and anxiety (14). However, numerous studies suggested that the prevalence of HTN validated by multiple BP measurements is considerably lower than that of elevated BP defined by a single visit in children and adolescents (15–17). A systematic review indicated that the overall prevalence of elevated BP in participants aged 3–20 decreased from 12.1% at the first visit to 5.6 and 2.7% at the second and third visits, respectively (18). Therefore, HTN assessment based on three separate BP visits is required for an accurate understanding of BP levels in children and adolescents, which have been recommended by the latest standards in many countries (19–21).

In general, adolescents with overweight and obesity were more susceptible to HTN than normal weight individuals, regardless of sex (22). It is well acknowledged that the increased prevalence of pediatric HTN couples with the obesity epidemic (23). Body mass index (BMI), a measurement based on a person’s height and weight, is the current screening standard for obesity. However, it has been revealed that BMI does not distinguish between fat mass and lean mass (24). Generally, weight is not proportional to height squared in children and adolescents, resulting in BMI instability (25, 26). To compensate for the significant changes in BMI during childhood, age-in-month cutoffs have been widely used (27). Nevertheless, the cutoffs are multiple and complex, with many age- and sex-specific thresholds.

Recently, Peterson et al. (28) found that tri-ponderal mass index (TMI, measured in kg/m^3^), an emerging indicator of obesity, may perform as a more valid obesity indicator to measure body fat in adolescents. Unlike BMI, TMI is relatively stable during adolescence, and its centile cutoffs fluctuate within a narrow range. Wang et al. have suggested a threshold with only four cutoffs to screen for overweight and obesity in children aged 7–18 years (29). Additionally, TMI has a lower overall misclassification rate than BMI in discriminating central obesity and HTN in overweight adolescents (30). Hence, TMI was considered a more reliable index for overweight and obesity (28–30). However, a systematic review (31) concluded that evidence of whether TMI outperforms BMI in identifying BP remains limited and inconsistent, while prospective reports are insufficient. In addition, whether TMI outperforms BMI in predicting HTN measured by three separate times is far from the conclusion.

Therefore, we used data through three separate BP visits from the Health Promotion Program for Children and Adolescents (HPPCA) in Suzhou of China to verify whether TMI has better accuracy than BMI in discriminating adolescent HTN and HTN subtypes.

## Materials and methods

### Study design and participants

This study was a retrospective cohort analysis based on HPPCA data for nine consecutive years (from 2012 to 2020). HPPCA is a large-scale ongoing school-based monitoring program conducted in Suzhou, China. Detailed information about HPPCA was published in the previous studies (32, 33). In brief, HPPCA provided free annual health check-ups for all school-based students aged 6–17 years in Suzhou to assess the growth and development of children and adolescents. Students are examined in general hospitals, centers for disease prevention and control, or community medical institutions. Students in the third year of junior high school or senior high school who would take other special physical examinations for school entrance were excluded from the study because their data are not currently accessible.

**FIGURE 1:**
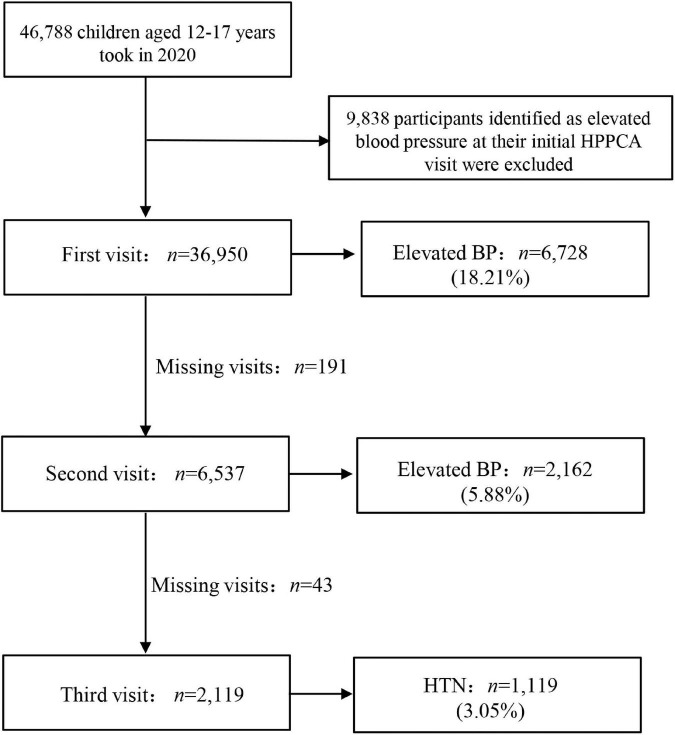
Details of the study population selection. HPPCA, health promotion program for children and adolescents; BP, blood pressure; HTN, hypertension.

To figure out the epidemic status of HTN among adolescents in Suzhou, we selected 66 junior or senior high schools attending HPPCA to conduct specific BP surveillance in 2020. All participants who had elevated BP in the first BP measurement were included in the second visit at least 2 weeks later; only those with elevated BP at the second visit were included in the third visit. To eliminate the “white-coat” effect, the second and third BP measurements were conducted by a familiar school nurse of the particular student, following the same criteria as the HPPCA. Almost all students with elevated BP at their first (follow-up rate: 97.16%) or second (follow-up rate: 98.01%) visit participated in the follow-up BP measurement, except for children who transferred to another school or took extended sick leave. The initial sample consisted of 46,788 school-based adolescents aged 12–17 years. To accurately portray the morbidity of HTN in adolescents, we used the 2020 surveillance data as an endpoint and excluded 9,838 children with elevated BP at their initial HPPCA visit from 2012 to 2019. Figure 1 shows the flowchart of the detailed study design, which was coherent with other large-scale surveillance studies in China (17, 34).

This study was approved by the Ethics Committee of the Suzhou Center for Disease Prevention and Control (No. SZJK2020-XW001). Written informed consent was obtained from all the participants or their guardians.

### Anthropometric measurements and weight status classification

All physical examinations were performed by well-trained health professionals using the same type of age-appropriate equipment and following the same procedures. Participants were requested to remove their shoes and wear light clothing before being measured to an accuracy of 0.1 cm (height) and 0.1 kg (weight). BMI is calculated as weight (kg) divided by the square of height (m), whereas TMI is calculated as weight (kg) divided by the cube of height (m).

Participants were classified as underweight, normal weight, overweight, and obesity according to the latest Chinese pediatric standards of age- and sex-specific BMI cutoffs (21) and the TMI criteria proposed by Wang et al. (29) on a Chinese population, respectively. Noteworthy, the TMI cutoffs to define adolescent overweight and obesity were 13.1 and 14.1 kg/m^3^ for participants under 16, respectively (29). The corresponding TMI cutoffs for those aged 16 or over were 14.0 and 15.8 kg/m3, respectively (29).

### Blood pressure measurements and definitions

The BP of children and adolescents was measured each visit on the right arm using a clinically validated Electronic Blood Pressure Monitor (i.e., Omron HBP1300, HBP1320) of appropriate size after a 15 min sit-down rest period in a quiet environment. The BP device was placed at the same level as the participant’s heart and right arm cuff. Three consecutive BP values were measured at 2-min intervals for each visit, and the average of the two closest BP readings was recorded for diagnosis and statistical analysis.

BP status was also categorized according to the Chinese standard “Reference of screening for elevated BP among children and adolescents aged 7∼18 years” (WS/T 610-2018) (21). Elevated BP was defined as systolic blood pressure (SBP), diastolic blood pressure (DBP), or both equal to or above the age-, sex-, and height-specific 95th percentile. Notably, HTN is diagnosed only when elevated BP is present at all three separate visits (21). Based on 3 separate visits, isolated systolic hypertension (ISH), isolated diastolic hypertension (IDH), and systolic and diastolic hypertension (SDH) was defined as SBP ≥ P95 and DBP < P95, DBP ≥ P95 and SBP < P95, and SBP ≥ P95 and DBP ≥ P95, respectively.

### Statistical analysis

The basic information about the participants was described in the total sample and by age and sex. Continuous variables were expressed as mean ± standard deviation (SD), and categorical variables were described as n (%). Student’s *t*-test and Chi-square test were used to compare differences between groups, respectively.

The AUC, the false-positive rate (FPR), the false-negative rate (FNR), and total misclassification rates were used to directly measure the discrimination ability between youth BMI and TMI. The net reclassification improvement (NRI) measures the correct movement in categories—upwards for events and downwards for non-events—using reclassification tables constructed separately for participants with and without events (35). We only obtained continuous NRI because no established NRI categories guide clinical decisions for HTN risk in Chinese children were found. The integrated discrimination improvement (IDI) essentially measures how the R^2^ (explained variance) improves when a new risk factor is introduced (36). Analyses were conducted using SAS statistical software (version 9.4, SAS Institute) and R (version 4.2.0, R Foundation for Statistical Computing). All reported *P*-values were two-tailed, and *P* < 0.05 was considered statistically significant.

## Results

### Baseline characteristics of the participants

The overall population consists of 36,950 adolescents aged 12–17 years from HPPCA in 2020. Table 1 demonstrates the primary characteristic of the overall participants. A total of 18,797 boys account for 50.87% of the overall population. The average age of the overall population was 14.39 (SD = 1.68) in 2020. Compared with girls, boys had higher BMI (21.42 vs. 20.45 kg/m^2^, *P* < 0.001). According to BMI cutoffs, the prevalence of overweight and obesity among adolescents in 2020 was 17.20 and 10.28%, respectively. According to TMI cutoffs, the corresponding prevalence was 13.27 and 18.97%. Comparisons between included and excluded populations from the first visit to the second visit (97.16% of cooperation rate) and the second visit to the third (98.01% of cooperation rate) are shown in Supplementary Tables 1, 2, respectively. The vast majority of the variable characteristics yielded no significant difference between included and excluded populations from the first visit to the second visit. A slight difference was found between included and excluded populations from the second visit to the third visit.

**TABLE 1:** Basic characteristics of Chinese adolescents.

Variables	Overall	Boys	Girls	*P*-value
			
	*n* = 36,950	*n* = 18,797	*n* = 18,153	
**Initial HPPCA visit**				
Age (year)	10.23 ± 2.77	10.21 ± 2.77	10.25 ± 2.77	0.169
Height (cm)	143.50 ± 17.21	144.50 ± 18.08	142.50 ± 16.19	<0.001
Weight (kg)	38.52 ± 14.20	40.04 ± 15.32	36.96 ± 12.74	<0.001
BMI (kg/m^2^)	18.06 ± 3.26	18.47 ± 3.44	17.62 ± 3.01	<0.001
TMI (kg/m^3^)	12.61 ± 1.94	12.82 ± 2.04	12.40 ± 1.80	<0.001
SBP (mmHg)	102.92 ± 11.65	104.10 ± 12.31	101.80 ± 10.80	<0.001
DBP (mmHg)	64.42 ± 7.10	64.29 ± 7.22	64.56 ± 6.97	<0.001
**In 2020**				
Age (year)	14.39 ± 1.68	14.36 ± 1.68	14.42 ± 1.69	0.001
Height (cm)	165.35 ± 8.65	169.30 ± 9.05	161.20 ± 5.84	<0.001
Weight (kg)	57.62 ± 12.71	61.83 ± 14.01	53.27 ± 9.40	<0.001
BMI (kg/m^2^)	20.94 ± 3.61	21.42 ± 3.92	20.45 ± 3.19	<0.001
TMI (kg/m^3^)	12.68 ± 2.15	12.66 ± 2.28	12.70 ± 2.01	0.091
**District**				
Urban	22,398 (60.62%)	11,403 (60.66%)	10,996 (60.57%)	0.863
Rural	14,552 (39.38%)	7,394 (39.34%)	7,157 (39.43%)	
**BMI status**				
Underweight	2,089 (5.65%)	1,255 (6.68%)	834 (4.59%)	<0.001
Normal weight	24,706 (66.86%)	10,858 (57.76%)	13,848 (76.28%)	
Overweight	6,357 (17.20%)	4,086 (21.74%)	2,271 (12.51%)	
Obesity	3,798 (10.28%)	2,598 (13.82%)	1,200 (6.61%)	
**TMI status**				
Underweight and normal	25,047 (67.79%)	12,518 (66.60%)	12,529 (69.02%)	<0.001
Overweight	4,893 (13.24%)	2,316 (12.32%)	2,577 (14.20%)	
Obesity	7,010 (18.97%)	3,963 (21.08%)	3,047 (16.79%)	
**SBP (mmHg)**				
First visit	114.32 ± 12.46	118.20 ± 12.24	110.30 ± 11.36	<0.001
Second visit	120.47 ± 13.16	123.90 ± 13.32	116.50 ± 11.77	<0.001
Third visit	126.13 ± 11.91	129.90 ± 11.51	121.70 ± 10.82	<0.001
*P* for trend	<0.001	<0.001	<0.001	
**DBP (mmHg)**				
First visit	68.55 ± 8.41	68.56 ± 8.66	68.53 ± 8.14	0.708
Second visit	72.91 ± 8.69	72.74 ± 8.88	73.10 ± 8.45	0.101
Third visit	75.32 ± 8.27	75.22 ± 8.44	75.43 ± 8.06	0.569
*P* for trend	<0.001	<0.001	<0.001	
**ISH (*n*, %)**				
First visit	4,103 (11.10%)	2,460 (13.09%)	1,643 (9.05%)	<0.001
Second visit	1,161 (3.16%)	729 (3.90%)	432 (2.39%)	<0.001
Third visit	658 (1.79%)	402 (2.15%)	256 (1.42%)	<0.001
*P* for trend	<0.001	<0.001	<0.001	
**IDH (*n*, %)**				
First visit	1,526 (4.13%)	690 (3.67%)	836 (4.61%)	<0.001
Second visit	408 (1.11%)	153 (0.82%)	255 (1.41%)	<0.001
Third visit	156 (0.42%)	53 (0.28%)	103 (0.57%)	<0.001
*P* for trend	<0.001	<0.001	<0.001	
**SDH (*n*, %)**				
First visit	1,099 (2.97%)	476 (2.53%)	623 (3.43%)	<0.001
Second visit	593 (1.61%)	280 (1.50%)	313 (1.73%)	0.073
Third visit	305 (0.83%)	139 (0.74%)	166 (0.92%)	0.063
*P* for trend	<0.001	<0.001	<0.001	
**Elevated BP (*n*, %)**				
First visit	6,728 (18.21%)	3,626 (19.29%)	3,102 (17.09%)	<0.001
Second visit	2,162 (5.88%)	1,162 (6.22%)	1,000 (5.53%)	0.909
Third visit (HTN)	1,119 (3.05%)	594 (3.18%)	525 (2.91%)	0.133
*P* for trend	<0.001	<0.001	<0.001	

HPPCA, Health Promotion Program for Children and Adolescents; BMI, body mass index; TMI, tri-ponderal mass index; SBP, systolic blood pressure; DBP, diastolic blood pressure; ISH, isolated systolic hypertension; IDH, isolated diastolic hypertension; SDH, systolic and diastolic hypertension; BP, blood pressure; HTN, hypertension.

After the three visits, the incidence of HTN, ISH, IDH, and SDH decreased to 3.05, 1.79, 0.42, and 0.83%, respectively. During the three visits, boys had higher SBP and prevalence of ISH than girls, while girls had a higher prevalence of IDH than boys (all *P* < 0.05).

### Analysis of the anthropometric indicators for predicting hypertension and hypertension subtypes

The comparison predictors (95% CI) of the discriminative ability between TMI and BMI for HTN and HTN subtypes are shown in Tables 2–5. Supplementary Tables 3–6 show detailed results performed by gender. Generally speaking, TMI had higher AUCs than BMI, and its NRIs significantly increased both in HTN (difference in AUC = 0.019, 95% CI = 0.007-0.031; NRI = 0.067, 95% CI = 0.008-0.127) and in ISH (difference in AUC = 0.021, 95% CI = 0.005–0.036; NRI = 0.106, 95% CI = 0.029–0.183).

**TABLE 2:** Comparison of anthropometric indices in predicting hypertension in Chinese adolescents.

	AUC (95%CI)				NRI (95%CI)		IDI (95%CI)	
			
	TMI	BMI	Difference	*P*-value	Difference	*P*-value	Difference	*P*-value
Total	0.645 (0.628, 0.662)	0.626 (0.608, 0.643)	0.019 (0.007, 0.031)	0.002	0.067 (0.008, 0.127)	0.027	0.000 (–0.000, 0.001)	0.424
**Sex**								
Boys	0.646 (0.623, 0.669)	0.645 (0.622, 0.669)	0.000 (–0.016, 0.017)	0.951	–0.023 (–0.105, 0.059)	0.579	–0.001 (–0.003, 0.000)	0.076
Girls	0.642 (0.617, 0.667)	0.602 (0.577, 0.628)	0.040 (0.021, 0.059)	<0.001	0.209 (0.123, 0.296)	<0.001	0.002 (0.001, 0.003)	<0.001
**Age**								
<16	0.634 (0.614, 0.655)	0.619 (0.598, 0.640)	0.015 (0.001, 0.029)	0.037	0.009 (–0.080, 0.063)	0.815	0.000 (–0.001, 0.000)	0.542
=16	0.673 (0.644, 0.703)	0.638 (0.607, 0.668)	0.036 (0.017, 0.055)	<0.001	0.352 (0.246, 0.458)	<0.001	0.006 (0.004, 0.008)	<0.001
**BMI**								
Underweight	0.507 (0.295, 0.720)	0.623 (0.485, 0.760)	–0.115 (–0.345, 0.114)	0.325	–0.173 (–0.763, 0.418)	0.566	0.000 (–0.002, 0.000)	0.165
Normal weight	0.530 (0.503, 0.556)	0.518 (0.491, 0.544)	0.012 (–0.013, 0.037)	0.357	0.084 (–0.006, 0.173)	0.067	0.000 (0.000, 0.000)	0.059
Overweight	0.537 (0.504, 0.570)	0.527 (0.494, 0.560)	0.010 (–0.020, 0.039)	0.518	0.039 (–0.078, 0.155)	0.518	0.000 (–0.000, 0.001)	0.112
Obesity	0.534 (0.500, 0.567)	0.557 (0.524, 0.590)	–0.023 (–0.047, 0.001)	0.060	–0.148 (–0.262, –0.034)	0.011	–0.003 (–0.004, –0.001)	<0.001
**TMI**								
Underweight and Normal	0.533 (0.505, 0.561)	0.532 (0.503, 0.560)	0.001 (–0.025, 0.027)	0.938	0.034 (–0.062, 0.130)	0.490	0.000 (–0.000, 0.000)	0.756
Overweight	0.507 (0.467, 0.547)	0.531 (0.491, 0.572)	–0.025 (–0.056, 0.007)	0.129	0.008 (–0.128, 0.144)	0.908	–0.000 (–0.001, 0.000)	0.072
Obesity	0.561 (0.533, 0.588)	0.565 (0.538, 0.592)	–0.004 (–0.023, 0.015)	0.670	–0.107 (–0.200, –0.014)	0.024	–0.001 (–0.002, 0.000)	0.048

AUC, area under the curve; CI, confidential interval; NRI, net reclassification index; IDI, integrated discrimination improvement; BMI, body mass index; TMI, tri-ponderal mass index.

**TABLE 3:** Comparison of anthropometric indices in predicting isolated systolic hypertension in Chinese adolescents.

	AUC (95%CI)				NRI (95%CI)		IDI (95%CI)	
			
	TMI	BMI	Difference	*P*-value	Difference	*P*-value	Difference	*P*-value
Total	0.649 (0.627, 0.671)	0.628 (0.606, 0.650)	0.021 (0.005, 0.036)	0.009	0.106 (0.029, 0.183)	0.007	0.000 (–0.000, 0.001)	0.071
**Sex**								
Boys	0.650 (0.623, 0.678)	0.645 (0.617, 0.673)	0.005 (–0.013, 0.024)	0.581	0.020 (–0.079, 0.119)	0.693	0.000 (–0.001, 0.001)	0.943
Girls	0.633 (0.598, 0.668)	0.589 (0.553, 0.624)	0.044 (0.017, 0.072)	0.001	0.318 (0.197, 0.440)	< 0.001	0.002 (0.000, 0.002)	<0.001
**Age**								
<16	0.643 (0.617, 0.668)	0.630 (0.604, 0.657)	0.012 (–0.005, 0.030)	0.163	0.013 (–0.078, 0.104)	0.781	–0.000 (–0.000, 0.000)	0.846
=16	0.666 (0.626, 0.706)	0.628 (0.587, 0.668)	0.039 (0.011, 0.066)	0.005	0.366 (0.223, 0.508)	<0.001	0.002 (0.004, 0.005)	<0.001
**BMI**								
Underweight	0.497 (0.020, 0.975)	0.525 (0.201, 0.850)	–0.028 (–0.693, 0.637)	0.934	0.378 (–0.689, 1.446)	0.487	0.000 (–0.000, 0.000)	0.856
Normal weight	0.528 (0.494, 0.563)	0.518 (0.483, 0.553)	0.011 (–0.023, 0.044)	0.537	0.082 (–0.036, 0.200)	0.171	0.000 (–0.000, 0.000)	0.490
Overweight	0.513 (0.471, 0.556)	0.499 (0.458, 0.539)	0.015 (–0.060, 0.090)	0.696	0.030 (–0.119, 0.180)	0.690	0.000 (–0.000, 0.000)	0.481
Obesity	0.543 (0.500, 0.585)	0.548 (0.507, 0.588)	–0.005 (–0.036, 0.026)	0.751	–0.038 (–0.181, 0.104)	0.597	–0.000 (–0.001, 0.000)	0.409
**TMI**								
Underweight and Normal	0.545 (0.508, 0.581)	0.542 (0.504, 0.579)	0.003 (–0.033, 0.039)	0.860	0.058 (–0.069, 0.184)	0.372	0.000 (–0.000, 0.000)	0.544
Overweight	0.496 (0.445, 0.546)	0.512 (0.462, 0.562)	–0.016 (–0.054, 0.022)	0.403	0.027 (–0.150, 0.204)	0.763	0.000 (–0.000, 0.000)	0.620
Obesity	0.562 (0.527, 0.598)	0.557 (0.523, 0.591)	0.005 (–0.019, 0.029)	0.679	–0.005 (–0.122, 0.123)	0.935	0.000 (–0.000, 0.001)	0.443

AUC, area under the curve; CI, confidential interval; BMI, body mass index; NRI, net reclassification index; IDI, integrated discrimination improvement; TMI, tri-ponderal mass index.

**TABLE 4:** Comparison of anthropometric indices in predicting isolated diastolic hypertension in Chinese adolescents.

	AUC (95%CI)				NRI (95%CI)*		IDI (95%CI)	
			
	TMI	BMI	Difference	*P*-value	Difference	*P*-value	Difference	*P*-value
Total	0.581 (0.535, 0.626)	0.554 (0.505, 0.603)	0.027 (–0.007, 0.060)	0.119	NA	NA	0.002 (0.000, 0.004)	0.006
**Sex**								
Boys	0.572 (0.493, 0.651)	0.584 (0.497, 0.670)	–0.012 (–0.066, 0.043)	0.675	NA	NA	0.002 (–0.000, 0.005)	0.178
Girls	0.601 (0.545, 0.658)	0.557 (0.499, 0.615)	0.044 (0.001, 0.087)	0.043	NA	NA	0.002 (0.000, 0.003)	0.004
**Age**								
<16	0.543 (0.490, 0.595)	0.533 (0.479, 0.587)	0.010 (–0.029, 0.048)	0.628	NA	NA	0.000 (–0.000, 0.002)	0.306
=16	0.687 (0.598, 0.776)	0.671 (0.576, 0.766)	0.016 (–0.031, 0.064)	0.496	NA	NA	0.008 (0.003, 0.014)	0.004
**BMI**								
Underweight	0.526 (0.133, 0.919)	0.655 (0.421, 0.889)	–0.129 (–0.610, 0.352)	0.599	–0.799 (–0.841, –0.757)	<0.001	–0.000 (–0.002, 0.000)	0.350
Normal weight	0.539 (0.479, 0.601)	0.498 (0.438, 0.558)	0.041 (–0.014, 0.095)	0.142	NA	NA	0.000 (0.000, 0.000)	0.067
Overweight	0.503 (0.408, 0.598)	0.549 (0.441, 0.658)	–0.047 (–0.136, 0.042)	0.302	NA	NA	–0.000 (–0.000, 0.000)	0.667
Obesity	0.523 (0.414, 0.632)	0.588 (0.479, 0.697)	–0.066 (–0.154, 0.023)	0.145	NA	NA	–0.000 (–0.002, 0.002)	0.682
**TMI**								
Underweight and Normal	0.522 (0.457, 0.586)	0.502 (0.438, 0.567)	0.020 (–0.035, 0.075)	0.484	NA	NA	0.000 (–0.000, 0.000)	0.228
Overweight	0.501 (0.403, 0.599)	0.490 (0.372, 0.608)	0.011 (–0.072, 0.094)	0.795	NA	NA	0.000 (–0.001, 0.000)	0.672
Obesity	0.534 (0.452, 0.617)	0.570 (0.481, 0.659)	–0.036 (–0.097, 0.025)	0.249	NA	NA	0.000 (–0.002, 0.002)	0.781

AUC, area under the curve; CI, confidential interval; NRI, net reclassification index; IDI, integrated discrimination improvement; BMI, body mass index; TMI, tri-ponderal mass index.

*NA, Not recognized because the model construction conditions are not met.

**TABLE 5:** Comparison of anthropometric indices in predicting systolic and diastolic hypertension in Chinese adolescents.

	AUC (95%CI)				NRI (95%CI)		IDI (95%CI)	
			
	TMI	BMI	Difference	*P*-value	Difference	*P*-value	Difference	*P*-value
Total	0.659 (0.627, 0.692)	0.648 (0.615, 0.682)	0.011 (–0.012, 0.035)	0.353	–0.067 (–0.180, 0.046)	0.244	–0.000 (–0.001, –0.000)	0.003
**Sex**								
Boys	0.652 (0.604, 0.701)	0.661 (0.614, 0.708)	–0.008 (–0.044, 0.027)	0.640	–0.074 (–0.241, 0.093)	0.386	–0.000 (–0.002, –0.000)	0.017
Girls	0.674 (0.630, 0.718)	0.645 (0.599, 0.692)	0.028 (–0.003, 0.059)	0.076	–0.042 (–0.195, 0.110)	0.586	–0.001 (–0.002, –0.000)	0.013
**Age**								
<16	0.666 (0.624, 0.708)	0.641 (0.597, 0.684)	0.025 (–0.006, 0.056)	0.109	–0.067 (–0.214, 0.079)	0.369	–0.000 (–0.000, –0.000)	0.095
ł16	0.671 (0.621, 0.720)	0.635 (0.581, 0.690)	0.035 (0.006, 0.064)	0.016	0.002 (0.001, 0.004)	<0.001	0.002 (0.001, 0.004)	<0.001
**BMI**								
Underweight	0.547 (0.172, 0.922)	0.662 (0.432, 0.891)	–0.114 (–0.362, 0.133)	0.365	–0.105 (-1.086, 0.876)	0.834	–0.000 (–0.001, 0.000)	0.375
Normal weight	0.523 (0.470, 0.576)	0.532 (0.478, 0.586)	–0.009 (–0.060, 0.043)	0.741	–0.033 (–0.214, 0.148)	0.723	0.000 (–0.000, 0.000)	0.964
Overweight	0.593 (0.532, 0.654)	0.567 (0.507, 0.628)	0.026 (–0.033, 0.084)	0.394	0.134 (–0.075, 0.344)	0.210	0.000 (–0.000, 0.001)	0.532
Obesity	0.487 (0.426, 0.548)	0.557 (0.498, 0.617)	–0.070 (–0.184, 0.044)	0.227	–0.174 (–0.376, 0.027)	0.090	–0.000 (–0.002, 0.000)	0.090
**TMI**								
Underweight and normal	0.512 (0.455, 0.570)	0.532 (0.472, 0.591)	–0.020 (–0.070, –0.031)	0.453	0.015 (–0.182, 0.211)	0.885	0.000 (–0.000, 0.000)	0.890
Overweight	0.531 (0.451, 0.612)	0.587 (0.511, 0.663)	–0.056 (–0.122, 0.011)	0.101	–0.167 (–0.418, 0.083)	0.189	–0.001 (–0.002, 0.000)	0.013
Obesity	0.558 (0.511, 0.606)	0.570 (0.521, 0.620)	0.012 (–0.047, 0.022)	0.492	–0.220 (–0.385, –0.000)	0.009	–0.000 (–0.001, –0.000)	0.015

AUC, area under the curve; CI, confidential interval; NRI, net reclassification index; IDI, integrated discrimination improvement; BMI, body mass index; TMI, tri-ponderal mass index.

When predicting ISH (difference in AUC = 0.039, 95% CI = 0.011–0.066; NRI = 0.366, 95% CI = 0.223–0.508; IDI = 0.002, 95% CI = 0.004–0.005) and SDH (difference in AUC = 0.035, 95% CI = 0.006–0.064; NRI = 0.002, 95% CI = 0.001–0.004; IDI = 0.002, 95% CI = 0.001–0.004), the difference between TMI and BMI were more notable in those with age ≥ 16. For girls, there was a statistically significant difference in the AUCs of TMI vs. BMI for discriminating HTN (difference in AUC = 0.040; 95% CI = 0.021–0.059; NRI = 0.209, 95% CI = 0.123–0.296; IDI = 0.002, 95% CI = 0.001–0.003), ISH (difference in AUC = 0.044; 95% CI, 0.017–0.072; NRI = 0.318, 95% CI = 0.197–0.440; IDI = 0.002, 95% CI = 0.000–0.002), and IDH (difference in AUC = 0.044; 95% CI, 0.001–0.087; IDI = 0.002, 95% CI = 0.000–0.003). No difference was found in the AUCs of TMI vs. BMI for HTN and its subtypes in boys.

**FIGURE 2:**
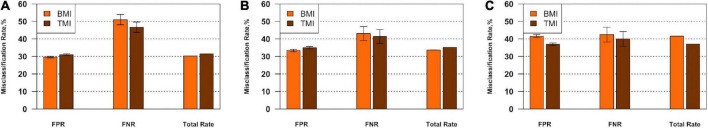
FPR, FNR, and total misclassification rates with 95% confidential intervals for TMI vs. BMI in predicting HTN in overall participants **(A)**, boys **(B)**, and girls **(C)** aged 12–17 years, respectively. BMI, body mass index; TMI, tri-ponderal mass index; FPR, false-positive rate; FNR, false-negative rate; HTN, hypertension.

As shown in Figure 2, TMI had lower FNR (*P* < 0.05) but higher FPR (*P* < 0.001) and total misclassification rates (*P* < 0.001) in predicting HTN for children compared with BMI. TMI had significantly lower FPR (*P* < 0.001) and total misclassification rates (*P* < 0.001) in girls as well as significantly higher FPR (*P* < 0.05) and total misclassification rates (*P* < 0.001) in boys. For boys, the FPR, FNR, and total misclassification rates for TMI were 35.0% (95% CI = 34.3–35.7%), 41.4% (95% CI = 37.5–45.4%) and 35.2% (95% CI = 35.2–35.2%), respectively; the corresponding rates for BMI were 33.4% (95% CI = 32.7–34.1%), 43.1% (95% CI = 39.1–47.1%) and 33.7% (95% CI = 33.7–33.7%), respectively. For girls, the FPR, FNR, and total misclassification rates for TMI were 37.0% (95% CI = 36.3–37.7%), 40.0% (95% CI = 35.8–44.2%) and 37.1% (95% CI = 37.1–37.1%), respectively; the corresponding rates for BMI were 41.5% (95% CI = 40.8–42.5%), 42.5% (95% CI = 38.2–46.7%) and 41.6% (95% CI = 41.6–41.6%), respectively. In total, the FPR, FNR, and total misclassification rates for TMI were 31.0% (95% CI = 30.6–31.5%), 46.7% (95% CI = 43.8–49.7%) and 31.5% (95% CI = 31.5–31.5%), respectively; the corresponding rates for BMI were 29.7% (95% CI = 29.2–30.1%), 51.0% (95% CI = 48.1–54.0%) and 30.3% (95% CI = 30.3–30.3%), respectively.

## Discussion

In this study, we assess the capacity of TMI and BMI to predict adolescent HTN and HTN subtypes. Our study found that TMI slightly outperformed BMI in predicting adolescent HTN and ISH. Additionally, TMI may have a stronger predictive power for HTN in girls, while no difference was observed in boys. Considering age, it showed higher discrimination power in predicting HTN for the subgroup of age ≥ 16 than its counterparts.

Several studies have reported a positive correlation between weight gain and BP levels in children and adolescents (17, 34, 37, 38). However, there were limited studies that compared the correlation of TMI and BMI with BP, as summarized in a systematic review (31). According to Wang et al. (39), TMI outperformed BMI in detecting children and adolescents with HTN in the Chinese population. A similar finding was obtained in Italian research (30), in which the total misclassification rate of TMI in predicting HTN in adolescents was around one-third of the BMI percentile. In a longitudinal analysis, the current study’s findings support that TMI had a better ability to predict teenage HTN than BMI. We hypothesized that this might be related to the fact that TMI in children and adolescents correlates with body fat percent equal to or better than BMI (28, 40, 41) and has a superior ability to predict central obesity (30).

When it comes to subtypes of adolescent HTN, we found that TMI outperformed BMI in predicting adolescent ISH. However, TMI and BMI did not differ in predicting IDH or SDH. This disparity can be explained because obesity primarily impacts central pulsatile hemodynamic alterations, which are directly related to increased SBP, but has little effect on DBP (42). Previous finding revealed that SBP increases more stable yearly from childhood to adulthood compared with DBP (43). Furthermore, adolescents with a higher BMI were reported to have higher left ventricular weight, aortic wave amplitude, and SBP than their normal-weight counterparts (44).

Interestingly, we discovered that TMI outperformed BMI when diagnosing adolescent HTN in girls but not in boys. In detail, TMI had lower rates of false positives and total misclassification than BMI in girls but had the opposite results in boys. The disparity could be attributed to hormonal and puberty differences between boys and girls (45, 46). A previous study found that TMI performed better in predicting obesity in girls (29). Therefore, TMI may also have better prediction performance on girls’ HTN. Additionally, we discovered that the difference between BMI and TMI for predicting ISH and SDH was more obvious in the subgroup of age ≥ 16, while such a difference was not found in the subgroup of age < 16. Wang et al. revealed that the accuracy of TMI classification of overweight and obesity increased along with age, especially after the age of 16 (29). As a result, this disparity could be that TMI’s predictive power increases with age and does not significantly outperform BMI until late adolescence.

According to a systematic review by Sun et al. (31), TMI can better discriminate central obesity and reflect body fat storage. Earlier studies (47) have also reported that an index based on cubic powers of height predicts obesity equal to or better than BMI. Thus, an increasing number of researchers (28–30) recommend the use of TMI to detect body fat in adolescents. Based on previous researches (30, 39), we assume that TMI has a better predictive power for adolescent BP than BMI. TMI cut-off values can be considered a satisfactory alternative indicator to screening for obesity risk in children and adolescents due to the low fluctuations in TMI with age. As proposed previously by Wang et al. (29), TMI cut-off values can significantly reduce the amount of computation and complexity required for overweight and obesity screening compared to previous BMI-based screening. These could assist more primary health care workers in effectively identifying and better preventing and controlling obesity.

The current study has many significant advantages. First, this study was a retrospective investigation employing a longitudinal methodology. Compared to previous cross-sectional studies (30, 39), the fluctuations in height, body fat percentage, and BP during adolescence were fully considered. To the best of our knowledge, among the studies comparing TMI with BMI on teenage HTN, the current study is the first to include all subtypes and employ BP effects at three visits, which significantly reduced the misdiagnosis rate of HTN. The second and third BP measures in this study were taken by known school nurses on the campus of the specific students, which could be advantageous in eliminating the “white-coat” effect. Moreover, the 2020 specific BP surveillance showed a good collaboration level, with 97.16% for the second and 98.01% for the third visit. Additionally, the large sample size and standard measurement data obtained from this study’s general population improve the conclusions’ robustness.

### Study limitations

First, this study recruited adolescents from Suzhou, a developed location in eastern China that is geographically restricted and not representative of other regional or ethnic groups. Second, the second or third BP visit was only for children with elevated BP diagnosed at the previous visit, as recommended by other large-scale surveillance studies (21). Children with BP < P_95_ at the first or second visit were not followed at the subsequent visit, which may underestimate the prevalence of HTN. Besides, not every child with BP ≥ P_95_ at the first or second visit was enrolled at the subsequent visit. However, the cooperation rates were around 98%, and the included group was generally representative of the overall population. And the subtle difference in characteristics in population from the second visit to the third visit could be attributed to increased variability from a smaller sample size during the third visit. Additionally, we were unable to compare the validity of all obesity indicators for predicting HTN because information such as waist circumference and the waist-to-height ratio was not collected.

## Conclusion

TMI was a more reliable index of adolescent HTN and ISH than BMI, although differences exist between age and sex stratums. However, there was a comparable performance in the prediction of IDH and SDH. In contrast to the complex BMI-for-age charts, the cutoffs for TMI are greatly simplified. Therefore, the application of TMI may promote the primary prevention of adolescent HTN and health management of children. In the future, results of our studies should be replicated in large cross-sectional and longitudinal studies in other racial and ethnic population. In addition, longer follow-up studies that continue into adulthood should be conducted to assess the relative merits of TMI and BMI in the prediction of HTN. Besides, future studies would verify TMI’s utility in clinical practice and eventually contribute to establishing an optimum standard of TMI.

## Data availability statement

The raw data supporting the conclusions of this article will be made available by the authors, without undue reservation.

## Ethics statement

The studies involving human participants were reviewed and approved by the Ethics Committee of Suzhou Center for Disease Prevention and Control. Written informed consent to participate in this study was provided by the participants’ legal guardian/next of kin.

## Author contributions

HY and AG contributed to the design and concept of the manuscript. JH and YZ were responsible for the analysis, interpretation of data, and manuscript drafting. DH, ZD, and BH organized the database. WG and HL performed the statistical analysis. HS and JY were responsible for the critical revision of the manuscript for intellectual content. All authors wrote the manuscript and had final approval of the submitted and published versions.

## References

[1] Mills KT Stefanescu A He J. The global epidemiology of hypertension. *Nat Rev Nephrol.* (2020) 16:223–37. 10.1038/s41581-019-0244-2 32024986 PMC7998524

[2] Song P Zhang Y Yu J Zha M Zhu Y Rahimi K Global prevalence of hypertension in children: A systematic review and meta-analysis. *JAMA Pediatr.* (2019) 173:1154–63. 10.1001/jamapediatrics.2019.3310 31589252 PMC6784751

[3] Ewald DR Haldeman Ph DL. Risk factors in adolescent hypertension. *Glob Pediatr Health.* (2016) 3:2333794x15625159. 10.1177/2333794x15625159 27335997 PMC4784559

[4] Hanevold C Waller J Daniels S Portman R Sorof J International Pediatric Hypertension Association. The effects of obesity, gender, and ethnic group on left ventricular hypertrophy and geometry in hypertensive children: A collaborative study of the international pediatric hypertension association. *Pediatrics.* (2004) 113:328–33. 10.1542/peds.113.2.328 14754945

[5] Yan Y Hou D Liu J Zhao X Cheng H Xi B Childhood body mass index and blood pressure in prediction of subclinical vascular damage in adulthood: Beijing blood pressure cohort. *J Hypertens.* (2017) 35:47–54. 10.1097/hjh.0000000000001118 27648721

[6] Li Y Haseler E Chowienczyk P Sinha MD. Haemodynamics of hypertension in children. *Curr Hypertens Rep.* (2020) 22:60. 10.1007/s11906-020-01044-2 32840715 PMC7447661

[7] Meng L Hou D Zhao X Hu Y Liang Y Liu J Cardiovascular target organ damage could have been detected in sustained pediatric hypertension. *Blood Press.* (2015) 24:284–92. 10.3109/08037051.2015.1049424 26024395

[8] Franks PW Hanson RL Knowler WC Sievers ML Bennett PH Looker HC. Childhood obesity, other cardiovascular risk factors, and premature death. *N Engl J Med.* (2010) 362:485–93. 10.1056/NEJMoa0904130 20147714 PMC2958822

[9] Bao W Threefoot SA Srinivasan SR Berenson GS. Essential hypertension predicted by tracking of elevated blood pressure from childhood to adulthood: The bogalusa heart study. *Am J Hypertens.* (1995) 8:657–65. 10.1016/0895-7061(95)00116-7 7546488

[10] Dong Y Song Y Zou Z Ma J Dong B Prochaska JJ. Updates to pediatric hypertension guidelines: Influence on classification of high blood pressure in children and adolescents. *J Hypertens.* (2019) 37:297–306. 10.1097/hjh.0000000000001903 30044314 PMC6365252

[11] Chen X Wang Y. Tracking of blood pressure from childhood to adulthood: A systematic review and meta-regression analysis. *Circulation.* (2008) 117:3171–80. 10.1161/CIRCULATIONAHA.107.730366 18559702 PMC3568631

[12] Pocock SJ Bakris G Bhatt DL Brar S Fahy M Gersh BJ. Regression to the mean in symplicity htn-3: Implications for design and reporting of future trials. *J Am Coll Cardiol.* (2016) 68:2016–25. 10.1016/j.jacc.2016.07.775 27788856

[13] Jurko A Jr. Minarik M Jurko T Tonhajzerova I. White coat hypertension in pediatrics. *Ital J Pediatr.* (2016) 42:4. 10.1186/s13052-016-0213-3 26786497 PMC4717664

[14] Schulte W Neus H Thönes M von Eiff AW. Basal blood pressure variability and reactivity of blood pressure to emotional stress in essential hypertension. *Basic Res Cardiol.* (1984) 79:9–16. 10.1007/bf01935802 6539592

[15] Cheung EL Bell CS Samuel JP Poffenbarger T Redwine KM Samuels JA. Race and Obesity in Adolescent Hypertension. *Pediatrics.* (2017) 139:e20161433. 10.1542/peds.2016-1433 28557717 PMC5404724

[16] Zhang Q Yang L Zhang Y Zhao M Liang Y Xi B. Hypertension prevalence based on three separate visits and its association with obesity among chinese children and adolescents. *Front Pediatr.* (2019) 7:307. 10.3389/fped.2019.00307 31396500 PMC6668215

[17] Dong J Dong H Yan Y Cheng H Zhao X Mi J Prevalence of hypertension and hypertension phenotypes after three visits in chinese Urban children. *J Hypertens.* (2021) 40:1270–7. 10.1097/HJH.0000000000002977 34285150

[18] Sun J Steffen LM Ma C Liang Y Xi B. Definition of pediatric hypertension: Are blood pressure measurements on three separate occasions necessary? *Hypertens Res.* (2017) 40:496–503. 10.1038/hr.2016.179 28077857

[19] National High Blood Pressure Education Program Working Group on High Blood Pressure in Children and Adolescents. The fourth report on the diagnosis, evaluation, and treatment of high blood pressure in children and adolescents. *Pediatrics.* (2004) 114:555–76. 15286277

[20] Flynn JT Kaelber DC Baker-Smith CM Blowey D Carroll AE Daniels SR Clinical practice guideline for screening and management of high blood pressure in children and adolescents. *Pediatrics.* (2017) 140:e20171904. 10.1542/peds.2017-1904 28827377

[21] Dong Y Ma J Song Y Dong B Wang Z Yang Z National blood pressure reference for chinese han children and adolescents aged 7 to 17 years. *Hypertension.* (2017) 70:897–906. 10.1161/HYPERTENSIONAHA.117.09983 28923902 PMC5722224

[22] Zhao W Mo L Pang Y. Hypertension in adolescents: The role of obesity and family history. *J Clin Hypertens.* (2021) 23:2065–70. 10.1111/jch.14381 34783422 PMC8696221

[23] Jiang SZ Lu W Zong XF Ruan HY Liu Y. Obesity and hypertension. *Exp Ther Med.* (2016) 12:2395–9. 10.3892/etm.2016.3667 27703502 PMC5038894

[24] Rothman KJ. Bmi-related errors in the measurement of obesity. *Int J Obes.* (2008) 32(Suppl. 3):S56–9. 10.1038/ijo.2008.87 18695655

[25] Weber DR Leonard MB Zemel BS. Body composition analysis in the pediatric population. *Pediatr Endocrinol Rev.* (2012) 10:130–9. 23469390 PMC4154503

[26] Cole TJ. Weight/heightp compared to weight/height2 for assessing adiposity in childhood: Influence of age and bone age on P during puberty. *Ann Hum Biol.* (1986) 13:433–51. 10.1080/03014468600008621 3800308

[27] Cole TJ Bellizzi MC Flegal KM Dietz WH. Establishing a standard definition for child overweight and obesity worldwide: International survey. *BMJ.* (2000) 320:1240. 10.1136/bmj.320.7244.1240 10797032 PMC27365

[28] Peterson CM Su H Thomas DM Heo M Golnabi AH Pietrobelli A Tri-ponderal mass index Vs body mass index in estimating body fat during adolescence. *JAMA Pediatr.* (2017) 171:629–36. 10.1001/jamapediatrics.2017.0460 28505241 PMC5710345

[29] Wang X Ma J Huang S Dong B Dong Y Yang Z Use of tri-ponderal mass index in predicting late adolescent overweight and obesity in children aged 7-18. *Front Nutr.* (2022) 9:785863. 10.3389/fnut.2022.785863 35387193 PMC8978718

[30] Malavazos AE Capitanio G Milani V Ambrogi F Matelloni IA Basilico S Tri-ponderal mass index Vs body mass index in discriminating central obesity and hypertension in adolescents with overweight. *Nutr Metab Cardiovasc Dis.* (2021) 31:1613–21. 10.1016/j.numecd.2021.02.013 33741212

[31] Sun J Yang R Zhao M Bovet P Xi B. Tri-ponderal mass index as a screening tool for identifying body fat and cardiovascular risk factors in children and adolescents: A systematic review. *Front Endocrinol.* (2021) 12:694681. 10.3389/fendo.2021.694681 34744995 PMC8566753

[32] Hu J Liu J Wang J Shen M Ge W Shen H Unfavorable progression of obesity in children and adolescents due to Covid-19 pandemic: A school-based survey in China. *Obesity.* (2021) 29:1907–15. 10.1002/oby.23276 34582110 PMC8661564

[33] Ge W Hu J Xiao Y Liang F Yi L Zhu R Covid-19 related childhood bmi increases in China: A health surveillance-based ambispective cohort analysis. *Am J Prev Med.* (2022) S0749-3797:227–226. 10.1016/j.amepre.2022.04.015 35688722 PMC9072804

[34] Liu K Li C Gong H Guo Y Hou B Chen L Prevalence and risk factors for hypertension in adolescents aged 12 to 17 years: A school-based study in China. *Hypertension.* (2021) 78:1577–85. 10.1161/HYPERTENSIONAHA.121.17300 34538102

[35] Pencina MJ D’Agostino RB Sr. D’Agostino RB Jr. Vasan RS Evaluating the added predictive ability of a new marker: From area under the roc curve to reclassification and beyond. *Stat Med.* (2008) 27:157–72. 10.1002/sim.2929 17569110

[36] Pencina MJ D’Agostino RB Sr. Steyerberg EW. Extensions of net reclassification improvement calculations to measure usefulness of new biomarkers. *Stat Med.* (2011) 30:11–21. 10.1002/sim.4085 21204120 PMC3341973

[37] Sánchez-Zamorano LM Salazar-Martinez E Anaya-Ocampo R Lazcano-Ponce E. Body mass index associated with elevated blood pressure in mexican school-aged adolescents. *Prev Med.* (2009) 48:543–8. 10.1016/j.ypmed.2009.03.009 19286003

[38] Montazeri P Fossati S Clemente DBP Cirugeda L Elosua R Fernández-Barrés S Early-childhood bmi trajectories in relation to preclinical cardiovascular measurements in adolescence. *J Dev Orig Health Dis.* (2022) 13:322–9. 10.1017/s2040174421000441 34308826

[39] Wang X Dong B Ma J Song Y Zou Z Arnold L. Role of tri-ponderal mass index in cardio-metabolic risk assessment in children and adolescents: Compared with body mass index. *Int J Obes.* (2020) 44:886–94. 10.1038/s41366-019-0416-y 31332274

[40] De Lorenzo A Romano L Di Renzo L Gualtieri P Salimei C Carrano E Triponderal mass index rather than body mass index: An indicator of high adiposity in italian children and adolescents. *Nutrition.* (2019) 60:41–7. 10.1016/j.nut.2018.09.007 30529185

[41] Woolcott OO Bergman RN. Relative fat mass as an estimator of whole-body fat percentage among children and adolescents: A cross-sectional study using nhanes. *Sci Rep.* (2019) 9:15279. 10.1038/s41598-019-51701-z 31649287 PMC6813362

[42] Wildman RP Mackey RH Bostom A Thompson T Sutton-Tyrrell K. Measures of obesity are associated with vascular stiffness in young and older adults. *Hypertension.* (2003) 42:468–73. 10.1161/01.Hyp.0000090360.78539.Cd 12953016

[43] Cui Y Zhang F Wang H Zhao L Song R Han M Temporal associations between tri-ponderal mass index and blood pressure in Chinese children: A cross-lag analysis. *Nutrients.* (2022) 14:1783. 10.3390/nu14091783 35565750 PMC9103659

[44] Pierce GL Pajaniappan M DiPietro A Darracott-Woei ASK Kapuku GK. Abnormal central pulsatile hemodynamics in adolescents with obesity: Higher aortic forward pressure wave amplitude is independently associated with greater left ventricular mass. *Hypertension.* (2016) 68:1200–7. 10.1161/hypertensionaha.116.07918 27620396 PMC5836470

[45] Chung S. Growth and puberty in obese children and implications of body composition. *J Obes Metab Syndr.* (2017) 26:243–50. 10.7570/jomes.2017.26.4.243 31089526 PMC6489471

[46] Xu T Zhu G Liu J Han S. Gender-specific prevalence and associated risk factors of high normal blood pressure and hypertension among multi-ethnic Chinese adolescents aged 8-18 years old. *Blood Press.* (2015) 24:189–95. 10.3109/08037051.2015.1025474 25830569

[47] Cândido AP Freitas SN Machado-Coelho GL. Anthropometric measurements and obesity diagnosis in schoolchildren. *Acta Paediatr.* (2011) 100:e120–4. 10.1111/j.1651-2227.2011.02296.x 21449923

