# Supplementary material for: Comparisons of tri-ponderal mass index and body mass index in discriminating hypertension at three separate visits in adolescents: A retrospective cohort study
Source: Front Nutr. 2022 Oct 17;9:1028861. doi: 10.3389/fnut.2022.1028861 (PMC9618711; doi:10.3389/fnut.2022.1028861)
Supplement: Supplementary file 1 [file Data_Sheet_1.docx]

Supplementary Material

| **Table S1.** Comparison between follow-up and missed populations from the first visit to the second visit**.** | | | |
| --- | --- | --- | --- |
| Variables | Followed-up | Missing | *P-value* |
|  | n=6,537 | n=191 |  |
| Initial HPPCA visit |  |  |  |
| Age (year) | 9.85±2.70 | 10.77±2.13 | <0.001 |
| Height (cm) | 141.80±17.16 | 148.10±15.60 | <0.001 |
| Weight (kg) | 38.92±12.26 | 42.33±13.42 | 0.002 |
| BMI (kg/m^2^) | 18.61±3.62 | 18.82±3.62 | 0.441 |
| TMI (kg/m^3^) | 13.13±2.09 | 12.77±2.62 | 0.020 |
| SBP (mmHg) | 104.30±11.69 | 99.31±12.03 | <0.001 |
| DBP (mmHg) | 65.20±7.14 | 66.74±7.43 | 0.004 |
| In 2020 |  |  |  |
| Age (year) | 14.25±1.70 | 13.92±1.30 | 0.008 |
| Height (cm) | 165.20±8.80 | 163.60±8.62 | 0.010 |
| Weight (kg) | 61.46±14.45 | 58.17±13.48 | 0.002 |
| BMI (kg/m^2^) | 22.34±4.12 | 21.58±4.02 | 0.642 |
| TMI (kg/m^3^) | 13.53±2.42 | 13.21±2.45 | 0.760 |
| District |  |  |  |
| Urban | 3766 (57.61%) | 33 (17.28%) | <0.001 |
| Rural | 2771 (42.39%) | 158 (82.72%) |  |
| BMI status |  |  |  |
| Underweight | 175 (2.68%) | 7 (3.66%) | 0.578 |
| Normal weight | 3613 (55.27%) | 112 (58.64%) |  |
| Overweight | 1463 (22.38%) | 37 (19.37%) |  |
| Obesity | 1286 (19.67%) | 35 (18.32%) |  |
| TMI status |  |  |  |
| Underweight and Normal | 3459 (52.91%) | 106 (55.50%) | 0.498 |
| Overweight | 1024 (15.66%) | 24 (12.57%) |  |
| Obesity | 2054 (31.42%) | 61 (31.94%) |  |
| SBP (mmHg) | 129.60.34±10.15 | 129.00±8.69 | 0.418 |
| DBP (mmHg) | 76.61±9.25 | 74.94±9.54 | 0.014 |
| ISH (n, %) | 3978 (60.85%) | 125 (65.45%) | 0.200 |
| IDH (n, %) | 1490 (22.79%) | 36 (18.85%) | 0.200 |
| SDH (n, %) | 1069 (16.35%) | 30 (15.71%) | 0.812 |
| HPPCA, Health Promotion Program for Children and Adolescents; BMI, body mass index; | | | |
| TMI, tri-ponderal mass index; SBP, systolic blood pressure; DBP, diastolic blood | | | |
| pressure; ISH, isolated systolic hypertension; IDH, isolated diastolic hypertension; | | | |
| SDH, systolic and diastolic hypertension; HTN, hypertension; BP, blood pressure. | | | |

| **Table S2.** Comparison between follow-up and missed populations from the second visit to the third visit. | | | |
| --- | --- | --- | --- |
| Variables | Followed-up | Missing | *P-value* |
|  | n=2,119 | n=43 |  |
| Initial HPPCA visit |  |  |  |
| Age (year) | 10.09±2.72 | 10.54±2.43 | 0.278 |
| Height (cm) | 143.60±17.24 | 146.20±15.51 | 0.320 |
| Weight (kg) | 41.34±15.98 | 41.01±12.70 | 0.894 |
| BMI (kg/m^2^) | 19.32±3.88 | 18.72±2.99 | 0.312 |
| TMI (kg/m^3^) | 13.46±2.23 | 12.85±1.87 | 0.077 |
| SBP (mmHg) | 105.80±11.97 | 107.30±9.78 | 0.436 |
| DBP (mmHg) | 66.08±7.20 | 66.53±7.34 | 0.698 |
| In 2020 |  |  |  |
| Age (year) | 14.56±1.65 | 14.88±1.55 | 0.210 |
| Height (cm) | 165.80±8.70 | 166.70±8.54 | 0.509 |
| Weight (kg) | 64.63±15.21 | 61.04±13.29 | 0.125 |
| BMI (kg/m^2^) | 23.33±4.37 | 21.86±3.74 | 0.028 |
| TMI (kg/m^3^) | 14.08±2.57 | 13.13±2.21 | 0.017 |
| District |  |  |  |
| Urban | 1120 (52.86%) | 20 (46.51%) | 0.410 |
| Rural | 999 (47.14%) | 23 (53.49%) |  |
| BMI status |  |  |  |
| Underweight | 42 (1.98%) | 3 (6.98%) | 0.032 |
| Normal weight | 1028 (48.51%) | 23 (53.49%) |  |
| Overweight | 505 (23.83%) | 12 (27.91%) |  |
| Obesity | 544 (25.67%) | 5 (11.63%) |  |
| TMI status |  |  |  |
| Underweight and Normal | 939 (44.31%) | 22 (51.16%) | 0.564 |
| Overweight | 374 (17.65%) | 8 (18.60%) |  |
| Obesity | 806 (38.04%) | 13 (30.23%) |  |
| SBP (mmHg) |  |  |  |
| First visit | 131.40±10.18 | 127.60±10.15 | 0.016 |
| Second visit | 132.50±10.66 | 127.60±9.97 | 0.003 |
| DBP (mmHg) |  |  |  |
| First visit | 77.41±8.98 | 78.28±8.87 | 0.528 |
| Second visit | 79.24±8.55 | 78.45±7.91 | 0.548 |
| ISH (n, %) |  |  |  |
| First visit | 1275 (60.17%) | 22 (51.16%) | 0.233 |
| Second visit | 1138 (53.70%) | 23 (53.49%) | 0.978 |
| IDH (n, %) |  |  |  |
| First visit | 405 (19.11%) | 12 (27.91%) | 0.148 |
| Second visit | 393 (18.55%) | 15 (34.88%) | 0.007 |
| SDH (n, %) |  |  |  |
| First visit | 439 (20.72%) | 9 (20.93%) | 0.973 |
| Second visit | 588 (27.75%) | 5 (11.63%) | 0.019 |
| HPPCA, Health Promotion Program for Children and Adolescents; BMI, body mass index; | | | |
| TMI, tri-ponderal mass index; SBP, systolic blood pressure; DBP, diastolic blood | | | |
| pressure; ISH, isolated systolic hypertension; IDH, isolated diastolic hypertension; | | | |
| SDH, systolic and diastolic hypertension; HTN, hypertension; BP, blood pressure. | | | |

| **Table S3**. Comparison of anthropometric indices in predicting hypertension in Chinese boys and girls. | | | | | | |  | |  | |  | |  | |  |
| --- | --- | --- | --- | --- | --- | --- | --- | --- | --- | --- | --- | --- | --- | --- | --- |
|  | AUC (95%CI) | |  |  |  | NRI (95%CI) | | | |  | | IDI (95%CI) | | | |
|  | TMI | BMI | Difference | *P-value* |  | Difference | | *P-value* | |  | | Difference | | *P-value* | |
| Boys | 0.646(0.623, 0.669) | 0.645(0.622, 0.669) | 0.000(-0.016, 0.017) | 0.951 |  | -0.023(-0.105, 0.059) | | 0.579 | |  | | -0.001(-0.003, 0.000) | | 0.076 | |
| Age |  |  |  |  |  |  | |  | |  | |  | |  | |
| <16 | 0.645(0.616, 0.674) | 0.649(0.620, 0.677) | -0.004(-0.022, 0.015) | 0.703 |  | -0.146(-0.245, -0.047) | | 0.004 | |  | | -0.002(-0.004, -0.000) | | 0.008 | |
| ≥16 | 0.668(0.631, 0.706) | 0.623(0.583, 0.663) | 0.045(0.019, 0.072) | <0.001 |  | 0.237(0.094, 0.381) | | 0.001 | |  | | 0.005(0.003, 0.007) | | <0.001 | |
| BMI |  |  |  |  |  |  | |  | |  | |  | |  | |
| Underweight | 0.577(0.329, 0.825) | 0.687(0.497, 0.877) | -0.110(-0.453, 0.232) | 0.528 |  | -0.237(-0.973, 0.498) | | 0.527 | |  | | -0.002(-0.006, 0.003) | | 0.468 | |
| Normal weight | 0.521(0.479, 0.563) | 0.546(0.505, 0.588) | -0.025(-0.065, 0.014) | 0.204 |  | -0.081(-0.220, 0.057) | | 0.248 | |  | | -0.000(-0.000, 0.000) | | 0.057 | |
| Overweight | 0.522(0.479, 0.565) | 0.527(0.485, 0.569) | -0.005(-0.043, 0.033) | 0.794 |  | -0.057(-0.205, 0.092) | | 0.456 | |  | | 0.000(-0.000, 0.000) | | 0.671 | |
| Obesity | 0.544(0.502, 0.586) | 0.568(0.527, 0.609) | -0.024(-0.056, 0.009) | 0.151 |  | -0.175(-0.319, -0.031) | | 0.017 | |  | | -0.003(-0.005, -0.001) | | <0.001 | |
| TMI |  |  |  |  |  |  | |  | |  | |  | |  | |
| Underweight and Normal | 0.539(0.500, 0.579) | 0.559(0.519, 0.599) | -0.020(-0.056, 0.017) | 0.291 |  | -0.043(-0.177, 0.091) | | 0.528 | |  | | -0.000(-0.000, 0.000) | | 0.043 | |
| Overweight | 0.512(0.455, 0.568) | 0.565(0.512, 0.618) | -0.054(-0.095, -0.012) | 0.012 |  | -0.118(-0.306, 0.071) | | 0.221 | |  | | -0.002(-0.004, -0.000) | | 0.013 | |
| Obesity | 0.558(0.521, 0.595) | 0.571(0.535, 0.606) | -0.012(-0.039, -0.014) | 0.363 |  | -0.203(-0.328, -0.078) | | 0.001 | |  | | -0.002(-0.003, -0.000) | | 0.048 | |
|  |  |  |  |  |  |  | |  | |  | |  | |  | |
| Girls | 0.642(0.617, 0.667) | 0.602(0.577, 0.628) | 0.040(0.021, 0.059) | <0.001 |  | 0.209(0.123, 0.296) | | <0.001 | |  | | 0.002(0.001, 0.003) | | <0.001 | |
| Age |  |  |  |  |  |  | |  | |  | |  | |  | |
| <16 | 0.627(0.598, 0.657) | 0.592(0.562, 0.622) | 0.035(0.013, 0.058) | 0.002 |  | 0.176(0.073, 0.279) | | <0.001 | |  | | 0.001(-0.000, 0.002) | | 0.001 | |
| ≥16 | 0.676(0.629, 0.722) | 0.647(0.598, 0.695) | 0.029(0.004, 0.055) | 0.025 |  | 0.454(0.298, 0.611) | | <0.001 | |  | | 0.008(0.004, 0.012) | | <0.001 | |
| BMI |  |  |  |  |  |  | |  | |  | |  | |  | |
| Underweight | 0.596(0.178, 1.00) | 0.518(0.335, 0.701) | 0.079(-0.508, 0.665) | 0.793 |  | 0.212(-0.770, 1.194) | | 0.672 | |  | | 0.001(-0.003, 0.005) | | 0.543 | |
| Normal weight | 0.536(0.502, 0.570) | 0.501(0.468, 0.535) | 0.035(0.003, 0.067) | 0.032 |  | 0.061(-0.056, 0.179) | | 0.306 | |  | | 0.000(0.000, 0.000) | | 0.040 | |
| Overweight | 0.560(0.508, 0.612) | 0.529(0.476, 0.582) | 0.031(-0.017, 0.078) | 0.206 |  | 0.267(0.080, 0.454) | | 0.005 | |  | | 0.001(-0.000, 0.002) | | 0.074 | |
| Obesity | 0.519(0.462, 0.577) | 0.548(0.492, 0.603) | -0.028(-0.064, 0.007) | 0.113 |  | -0.159(-0.345, -0.027) | | 0.094 | |  | | -0.003(-0.005, -0.000) | | 0.031 | |
| TMI |  |  |  |  |  |  | |  | |  | |  | |  | |
| Underweight and Normal | 0.525(0.486, 0.564) | 0.505(0.465, 0.544) | 0.020(-0.017, 0.058) | 0.290 |  | -0.012(-0.149, 0.124) | | 0.858 | |  | | 0.000(0.000, 0.000) | | 0.087 | |
| Overweight | 0.494(0.438, 0.549) | 0.520(0.461, 0.577) | -0.025(-0.127, 0.076) | 0.623 |  | -0.025(-0.222, 0.173) | | 0.806 | |  | | -0.000(-0.000, 0.000) | | 0.673 | |
| Obesity | 0.570(0.529, 0.610) | 0.560(0.518, 0.602) | 0.010(-0.018, 0.037) | 0.501 |  | 0.031(-0.107, 0.169) | | 0.659 | |  | | 0.000(-0.001, 0.002) | | 0.789 | |
| AUC, area under the curve; CI, confidential interval; NRI, net reclassification index; IDI, integrated discrimination improvement; BMI, body mass index; TMI, tri-ponderal mass index. | | | | | | | | | | | | | | | |

| **Table S4**. Comparison of anthropometric indices in predicting isolated systolic hypertension in Chinese boys and girls. | | | | | | |  |  |  |  |
| --- | --- | --- | --- | --- | --- | --- | --- | --- | --- | --- |
|  | AUC (95%CI) | |  |  |  | NRI (95%CI) | |  | IDI (95%CI) | |
|  | TMI | BMI | Difference | *P-value* |  | Difference | *P-value* |  | Difference | *P-value* |
| Boys | 0.650(0.623, 0.678) | 0.645(0.617, 0.673) | 0.005(-0.013, 0.024) | 0.581 |  | 0.020(-0.079, 0.119) | 0.693 |  | 0.000(-0.001, 0.001) | 0.943 |
| Age |  |  |  |  |  |  |  |  |  |  |
| <16 | 0.646(0.613, 0.680) | 0.649(0.615, 0.683) | -0.002(-0.023, 0.018) | 0.822 |  | -0.114(-0.232, -0.005) | 0.059 |  | -0.001(-0.003, 0.000) | 0.147 |
| ≥16 | 0.673(0.626, 0.720) | 0.628(0.579, 0.677) | 0.045(0.011, 0.080) | 0.010 |  | 0.291(0.111, 0.471) | 0.002 |  | 0.003(0.002, 0.005) | <0.001 |
| BMI |  |  |  |  |  |  |  |  |  |  |
| Underweight | 0.518(0.054, 0.982) | 0.542(0.203, 0.882) | -0.024(-0.695, 0.647) | 0.943 |  | 0.354(-0.714, 1.422) | 0.516 |  | 0.000(-0.000, 0.000) | 0.940 |
| Normal weight | 0.530(0.479, 0.581) | 0.547(0.495, 0.598) | -0.017(-0.064, 0.030) | 0.488 |  | -0.080(-0.247, 0.087) | 0.348 |  | -0.000(-0.000, 0.000) | 0.073 |
| Overweight | 0.507(0.455, 0.558) | 0.506(0.456, 0.556) | 0.000(-0.041, 0.043) | 0.974 |  | -0.019(-0.197, 0.160) | 0.838 |  | 0.000(-0.000, 0.000) | 0.508 |
| Obesity | 0.553(0.504, 0.601) | 0.564(0.516, 0.612) | -0.012(-0.050, 0.028) | 0.564 |  | -0.156(-0.327, 0.016) | 0.076 |  | -0.001(-0.003, 0.000) | 0.082 |
| TMI |  |  |  |  |  |  |  |  |  |  |
| Underweight and Normal | 0.552(0.503, 0.600) | 0.564(0.514, 0.613) | -0.012(-0.057, 0.033) | 0.615 |  | -0.042(-0.207, 0.122) | 0.613 |  | -0.000(-0.000, 0.000) | 0.081 |
| Overweight | 0.492(0.426, 0.557) | 0.537(0.474, 0.600) | -0.045(-0.091, 0.000) | 0.052 |  | -0.072(-0.300, 0.157) | 0.537 |  | -0.000(-0.001, 0.000) | 0.294 |
| Obesity | 0.560(0.516, 0.604) | 0.565(0.523, 0.607) | -0.005(-0.036, -0.026) | 0.745 |  | -0.164(-0.312, -0.015) | 0.031 |  | -0.000(-0.002, 0.000) | 0.301 |
|  |  |  |  |  |  |  |  |  |  |  |
| Girls | 0.633(0.598, 0.668) | 0.589(0.553, 0.624) | 0.044(0.017, 0.072) | 0.001 |  | 0.318(0.197, 0.440) | <0.001 |  | 0.002(0.000, 0.002) | <0.001 |
| Age |  |  |  |  |  |  |  |  |  |  |
| <16 | 0.627(0.587, 0.666) | 0.593(0.553, 0.634) | 0.033(0.002, 0.064) | 0.035 |  | 0.212(0.069, 0.355) | 0.004 |  | 0.001(0.000, 0.002) | 0.001 |
| ≥16 | 0.645(0.573, 0.716) | 0.604(0.533, 0.674) | 0.041(-0.003, 0.084) | 0.065 |  | 0.547(0.317, 0.777) | <0.001 |  | 0.005(0.001, 0.008) | 0.004 |
| BMI |  |  |  |  |  |  |  |  |  |  |
| Underweight | NA | NA | NA | NA |  | NA | NA |  | NA | NA |
| Normal weight | 0.526(0.480, 0.573) | 0.510(0.464, 0.556) | 0.016(-0.063, 0.096) | 0.685 |  | 0.041(-0.126, 0.207) | 0.632 |  | 0.000(-0.000, 0.000) | 1.000 |
| Overweight | 0.532(0.458, 0.606) | 0.511(0.439, 0.583) | 0.021(-0.108, 0.151) | 0.747 |  | -0.037(-0.308, 0.235) | 0.791 |  | 0.000(-0.000, 0.000) | 0.819 |
| Obesity | 0.518(0.434, 0.602) | 0.508(0.432, 0.584) | 0.011(-0.037, 0.058) | 0.662 |  | 0.006(-0.247, -0.259) | 0.963 |  | 0.000(-0.000, 0.002) | 0.281 |
| TMI |  |  |  |  |  |  |  |  |  |  |
| Underweight and Normal | 0.525(0.470, 0.579) | 0.499(0.444, 0.555) | 0.025(-0.068, 0.118) | 0.593 |  | -0.130(-0.323, 0.062) | 0.185 |  | 0.000(-0.000, 0.000) | 0.581 |
| Overweight | 0.533(0.455, 0.611) | 0.565(0.491, 0.639) | -0.032(-0.101, 0.038) | 0.371 |  | -0.207(-0.487, 0.073) | 0.147 |  | -0.000(-0.002, 0.000) | 0.120 |
| Obesity | 0.551(0.492, 0.610) | 0.528(0.472, 0.585) | 0.023(-0.016, 0.061) | 0.243 |  | 0.213(0.022, 0.405) | 0.029 |  | 0.002(0.000, 0.003) | 0.017 |
| AUC, area under the curve; CI, confidential interval; BMI, body mass index; NRI, net reclassification index; IDI, integrated discrimination improvement; TMI, tri-ponderal mass index. | | | | | | | | | | |

| **Table S5**. Comparison of anthropometric indices in predicting isolated diastolic hypertension in Chinese boys and girls. | | | | | | |  |  |  |  |
| --- | --- | --- | --- | --- | --- | --- | --- | --- | --- | --- |
|  | AUC (95%CI) | |  |  |  | NRI (95%CI) * | |  | IDI (95%CI) | |
|  | TMI | BMI | Difference | *P-value* |  | Difference | *P-value* |  | Difference | *P-value* |
| Boys | 0.572(0.493, 0.651) | 0.584(0.497, 0.670) | -0.012(-0.066, 0.043) | 0.675 |  | NA | NA |  | 0.002(-0.000, 0.005) | 0.178 |
| Age |  |  |  |  |  |  |  |  |  |  |
| <16 | 0.559(0.463, 0.655) | 0.605(0.507, 0.703) | -0.046(-0.116, 0.024) | 0.199 |  | NA | NA |  | 0.001(-0.002, 0.005) | 0.368 |
| ≥16 | 0.606(0.455, 0.756) | 0.535(0.336, 0.734) | 0.071(-0.014, 0.156) | 0.101 |  | NA | NA |  | 0.004(-0.005, 0.014) | 0.380 |
| BMI |  |  |  |  |  |  |  |  |  |  |
| Underweight | 0.687(0.083, 1.000) | 0.784(0.415, 1.000) | -0.097(-1.069, 0.875) | 0.845 |  | -0.579(-1.965, 0.808) | 0.413 |  | -0.002(-0.009, 0.004) | 0.484 |
| Normal weight | 0.496(0.373, 0.620) | 0.515(0.390, 0.640) | -0.019(-0.119, 0.082) | 0.714 |  | NA | NA |  | 0.000(-0.000, 0.000) | 0.723 |
| Overweight | 0.585(0.476, 0.693) | 0.527(0.354, 0.701) | 0.057(-0.178, 0.293) | 0.633 |  | NA | NA |  | -0.000(-0.001, 0.000) | 0.034 |
| Obesity | 0.520(0.363, 0.678) | 0.593(0.441, 0.745) | -0.073(-0.202, 0.057) | 0.271 |  | NA | NA |  | -0.000(-0.004, 0.003) | 0.801 |
| TMI |  |  |  |  |  |  |  |  |  |  |
| Underweight and Normal | 0.515(0.405, 0.625) | 0.523(0.406, 0.641) | -0.008(-0.092, 0.076) | 0.846 |  | NA | NA |  | 0.000(-0.000, 0.000) | 0.781 |
| Overweight | 0.503(0.371, 0.635) | 0.634(0.456, 0.812) | -0.131(-0.396, 0.134) | 0.332 |  | NA | NA |  | 0.000(-0.003, 0.004) | 0.753 |
| Obesity | 0.544(0.397, 0.691) | 0.588(0.434, 0.742) | -0.044(-0.152, -0.065) | 0.429 |  | NA | NA |  | -0.002(-0.003, -0.000) | 0.048 |
|  |  |  |  |  |  |  |  |  |  |  |
| Girls | 0.601(0.545, 0.658) | 0.557(0.499, 0.615) | 0.044(0.001, 0.087) | 0.043 |  | NA | NA |  | 0.002(0.000, 0.003) | 0.004 |
| Age |  |  |  |  |  |  |  |  |  |  |
| <16 | 0.558(0.494, 0.623) | 0.522(0.459, 0.584) | 0.037(-0.010, 0.084) | 0.122 |  | NA | NA |  | 0.000(-0.000, 0.002) | 0.214 |
| ≥16 | 0.735(0.625, 0.846) | 0.760(0.669, 0.850) | -0.024(-0.085, 0.036) | 0.431 |  | -0.086(-0.253, 0.081) | 0.311 |  | 0.004(0.000, 0.009) | 0.052 |
| BMI |  |  |  |  |  |  |  |  |  |  |
| Underweight | 0.747(0.308, 1.000) | 0.519(0.240, 0.799) | 0.228(-0.490, 0.946) | 0.533 |  | NA | NA |  | 0.000(-0.000, 0.000) | 0.815 |
| Normal weight | 0.556(0.484, 0.628) | 0.502(0.435, 0.569) | 0.054(-0.009, 0.117) | 0.092 |  | NA | NA |  | 0.000(0.000, 0.000) | 0.030 |
| Overweight | 0.548(0.414, 0.682) | 0.568(0.428, 0.708) | -0.020(-0.118, 0.078) | 0.688 |  | NA | NA |  | -0.000(-0.002, 0.000) | 0.279 |
| Obesity | 0.534(0.380, 0.688) | 0.603(0.441, 0.765) | -0.069(-0.195, 0.057) | 0.283 |  | NA | NA |  | -0.001(-0.003, 0.000) | 0.230 |
| TMI |  |  |  |  |  |  |  |  |  |  |
| Underweight and Normal | 0.535(0.454, 0.616) | 0.507(0.431, 0.583) | 0.028(-0.044, 0.099) | 0.446 |  | NA | NA |  | 0.000(0.000, 0.000) | 0.119 |
| Overweight | 0.526(0.402, 0.650) | 0.538(0.399, 0.677) | -0.012(-0.257, 0.233) | 0.924 |  | NA | NA |  | -0.000(-0.002, 0.000) | 0.434 |
| Obesity | 0.562(0.463, 0.662) | 0.586(0.475, 0.698) | -0.024(-0.101, 0.054) | 0.546 |  | NA | NA |  | 0.000(-0.001, 0.002) | 0.799 |
| AUC, area under the curve; CI, confidential interval; NRI, net reclassification index; IDI, integrated discrimination improvement; BMI, body mass index; TMI, tri-ponderal mass index.  *NA, Not recognized because the model construction conditions are not met. | | | | | | | | | | |

| **Table S6**. Comparison of anthropometric indices in predicting systolic and diastolic hypertension in Chinese boys and girls. | | | | | | | |  |  |  |
| --- | --- | --- | --- | --- | --- | --- | --- | --- | --- | --- |
|  | AUC (95%CI) | |  |  |  | NRI (95%CI) | |  | IDI (95%CI) | |
|  | TMI | BMI | Difference | *P-value* |  | Difference | *P-value* |  | Difference | *P-value* |
| Boys | 0.652(0.604, 0.701) | 0.661(0.614, 0.708) | -0.008(-0.044, 0.027) | 0.640 |  | -0.074(-0.241, 0.093) | 0.386 |  | -0.000(-0.002, -0.000) | 0.017 |
| Age |  |  |  |  |  |  |  |  |  |  |
| <16 | 0.674(0.609, 0.739) | 0.660(0.595, 0.725) | 0.014(-0.030, 0.058) | 0.536 |  | -0.161(-0.382, 0.059) | 0.151 |  | -0.000(-0.001, 0.000) | 0.122 |
| ≥16 | 0.662(0.594, 0.730) | 0.625(0.553, 0.697) | 0.037(-0.001, 0.083) | 0.118 |  | 0.184(-0.070, 0.437) | 0.157 |  | 0.002(0.000, 0.003) | 0.021 |
| BMI |  |  |  |  |  |  |  |  |  |  |
| Underweight | 0.554(0.096, 1.000) | 0.804(0.555, 1.000) | -0.251(-0.460, -0.041) | 0.019 |  | -1.331(-1.384, -1.279) | <0.001 |  | -0.002(-0.004, 0.001) | 0.231 |
| Normal weight | 0.505(0.414, 0.595) | 0.562(0.478, 0.645) | -0.057(-0.151, 0.037) | 0.237 |  | -0.170(-0.472, 0.132) | 0.269 |  | -0.000(-0.000, 0.000) | 0.462 |
| Overweight | 0.586(0.496, 0.676) | 0.578(0.496, 0.660) | 0.008(-0.074, 0.090) | 0.844 |  | -0.085(-0.372, 0.203) | 0.563 |  | -0.000(-0.001, 0.000) | 0.615 |
| Obesity | 0.522(0.433, 0.610) | 0.561(0.475, 0.646) | -0.039(-0.101, 0.023) | 0.216 |  | -0.230(-0.512, 0.051) | 0.109 |  | -0.000(-0.002, 0.000) | 0.160 |
| TMI |  |  |  |  |  |  |  |  |  |  |
| Underweight and Normal | 0.514(0.428, 0.601) | 0.565(0.482, 0.649) | -0.051(-0.135, 0.033) | 0.231 |  | -0.085(-0.378, 0.207) | 0.568 |  | 0.000(-0.000, 0.000) | 0.481 |
| Overweight | 0.561(0.434, 0.688) | 0.610(0.500, 0.720) | -0.049(-0.144, 0.045) | 0.305 |  | -0.242(-0.594, 0.111) | 0.179 |  | -0.002(-0.004, -0.000) | 0.040 |
| Obesity | 0.550(0.476, 0.623) | 0.572(0.501, 0.644) | -0.023(-0.078, 0.033) | 0.426 |  | -0.317(-0.562, -0.072) | 0.011 |  | -0.000(-0.001, 0.000) | 0.065 |
|  |  |  |  |  |  |  |  |  |  |  |
| Girls | 0.674(0.630, 0.718) | 0.645(0.599, 0.692) | 0.028(-0.003, 0.059) | 0.076 |  | -0.042(-0.195, 0.110) | 0.586 |  | -0.001(-0.002, -0.000) | 0.013 |
| Age |  |  |  |  |  |  |  |  |  |  |
| <16 | 0.674(0.619, 0.730) | 0.639(0.581, 0.697) | 0.035(-0.008, 0.078) | 0.107 |  | 0.010(-0.186, 0.205) | 0.922 |  | 0.000(-0.001, 0.000) | 0.229 |
| ≥16 | 0.680(0.608, 0.752) | 0.645(0.564, 0.726) | 0.035(0.001, 0.068) | 0.044 |  | 0.484(0.245, 0.724) | <0.001 |  | 0.003(0.000, 0.005) | 0.013 |
| BMI |  |  |  |  |  |  |  |  |  |  |
| Underweight | 0.555(0.000, 1.000) | 0.517(0.170, 0.863) | 0.039(-0.443, 0.520) | 0.876 |  | -0.075(-1.462, 1.313) | 0.916 |  | 0.000(-0.001, 0.002) | 0.779 |
| Normal weight | 0.535(0.469, 0.601) | 0.521(0.451, 0.590) | 0.014(-0.045, 0.074) | 0.636 |  | 0.016(-0.210, 0.242) | 0.891 |  | 0.000(-0.000, 0.000) | 0.626 |
| Overweight | 0.595(0.512, 0.679) | 0.558(0.469, 0.647) | 0.037(-0.047, 0.121) | 0.387 |  | 0.24(-0.068, 0.548) | 0.127 |  | 0.000(-0.000, 0.002) | 0.268 |
| Obesity | 0.514(0.429, 0.599) | 0.578(0.492, 0.664) | -0.064(-0.115, -0.013) | 0.013 |  | -0.432(-0.709, -0.156) | 0.002 |  | -0.003(-0.005, 0.000) | 0.088 |
| TMI |  |  |  |  |  |  |  |  |  |  |
| Underweight and Normal | 0.515(0.437, 0.594) | 0.510(0.428, 0.592) | 0.005(-0.056, 0.067) | 0.862 |  | 0.073(-0.192, 0.337) | 0.590 |  | 0.000(-0.000, 0.000) | 0.599 |
| Overweight | 0.513(0.416, 0.610) | 0.568(0.460, 0.675) | -0.055(-0.155, 0.045) | 0.281 |  | -0.275(-0.625, 0.074) | 0.123 |  | -0.000(-0.001, 0.000) | 0.211 |
| Obesity | 0.589(0.527, 0.652) | 0.587(0.518, 0.656) | 0.003(-0.042, 0.047) | 0.910 |  | -0.101(-0.322, 0.120) | 0.370 |  | -0.001(-0.002, 0.000) | 0.073 |
| AUC, area under the curve; CI, confidential interval; NRI, net reclassification index; IDI, integrated discrimination improvement; BMI, body mass index; TMI, tri-ponderal mass index. | | | | | | | | | | |
